# The ER glycoprotein folding sensor UDP-Glc: glycoprotein glucosyltransferase is broadly expressed in *C. elegans* hermaphrodite

**DOI:** 10.17912/micropub.biology.000299

**Published:** 2020-08-25

**Authors:** Lucila Buzzi, Victoria Ayelen Segobia, Diego Rayes, Olga A Castro

**Affiliations:** 1 Fundación Instituto Leloir, Buenos Aires, Argentina; 2 Departamento de Fisiología, Biología Molecular y Celular, Facultad de Ciencias Exactas y Naturales, Universidad de Buenos Aires, Buenos Aires, Argentina; 3 Instituto de Investigaciones Bioquímicas de Bahía Blanca (CONICET); 4 Departamento de Biología, Bioquímica y Farmacia, Universidad Nacional del Sur, Bahía Blanca, Argentina.; 5 Consejo Nacional de Investigaciones Científicas y Técnicas; 6 Instituto de Biociencias, Biotecnología y Biología traslacional, Departamento de Fisiología, Biología Molecular y Celular, Facultad de Ciencias Exactas y Naturales, Universidad de Buenos Aires, Buenos Aires, Argentina

**Figure 1. UGGT-1 expression in adult hermaphrodite:**
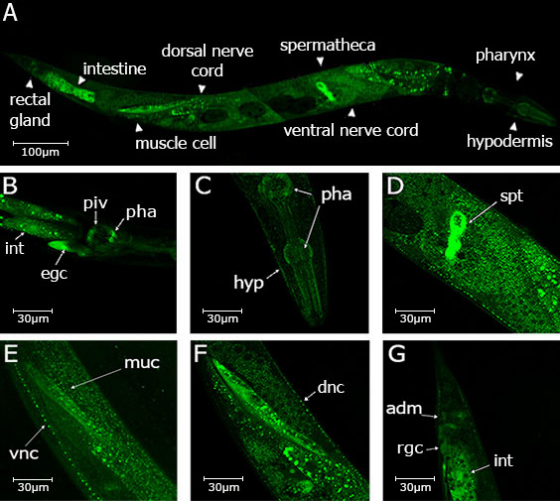
Panel A: full picture of an adult hermaphrodite constructed from images of several mosaic worms carrying the *exaEx101* extrachromosomal array, panels B-G depict magnified images of different parts of the hermaphrodite. Labels stand for the following: pha (pharynx); hyp (hypodermis); piv (pharyngeal-intestinal valve); int (intestine); egc (excretory gland cell), dnc (dorsal nerve cord), vnc (ventral nerve cord); rgc (rectal gland cell); spt (spermatheca); adm (anal depressor muscle).

## Description

The endoplasmic reticulum (ER) uses an elaborate system called the ER quality control (QC) to monitor the proper folding of newly synthesized glycoproteins. The QC allows cells to differentiate between properly folded and misfolded proteins, allowing only those proteins which have acquired their native conformations to exit the ER and reach their final destinations. Alternatively, misfolded glycoproteins or incompletely formed glycoprotein complexes are translocated to the cytosol where they are finally degraded by proteasomes (Caramelo and Parodi 2007). The key element of this mechanism is the UDP-Glc: glycoprotein glucosyltransferase (UGGT) that functions as a folding sensor as it glucosylates exclusively those glycoproteins that have not acquired their native structures (Trombetta **et al.*,* 1989; Caramelo **et al.*,* 2003, 2004). Only vertebrates and *Caenorhabditis* genomes carry two *uggt* gene copies (*uggt*–*1* and *uggt*–*2*) and phylogenetic inference showed that *uggt* genes went through independent duplications in *Caenorhabditis* and vertebrates. UGGT-1 retained canonical UGGT activity both in vertebrates and *Caenorhabditis* and vertebrate UGGT-2 underwent a specialization process. In *Caenorhabditis*, *uggt*-2 evolved by means of a putative neofunctionalization process in a non-redundant paralog and its biological function is still unknown (Caraballo **et al.*,* 2020; Buzzi *et al.*., 2011). Hence, UGGT-1 is the only protein engaged in monitoring the folding state of every glycoprotein in *Caenorhabditis* ER. To determine *C. elegans* UGGT-1’s body pattern expression we used fosmid recombineering technology (Tursun *et al.*, 2009) to generate the Puggt-1::sl2::nls::gfp::unc-54 3’UTR transcriptional fusion reporter and established worm lines expressing this construct. UGGT-1 is expressed in the head, both in the pharynx, (corpus, isthmus and terminal bulb and buccal cavity) and in the pharyngeal intestinal valve. In the same image its expression is detected in the hypodermis and in the secretory gland (B and C). The somatic cells of the spermatheca express UGGT-1, but not the germline (D). Consistent with our previous findings (Buzzi *et al.*., 2011) UGGT-1 is widely expressed in the nervous system, both in ventral and dorsal nerve cords (E and F), as well as in the muscle cells as shown in (E-F) and in the anal depressor muscle (G). In the tail expression is also observed both in the rectal gland cell and the intestine.

## Methods

Worms of stable transgenic lines carrying the *exaEx101* [Puggt-1::sl2::nls::gfp::unc-54 3’UTR] transcriptional fusion reporter were visualized by fluorescence confocal microscopy using an LSM510 Meta confocal microscope (Carl Zeiss, Oberkochen, Germany). Images were acquired with LSM software (Carl Zeiss) using a 20 x plan apochromat objective.
